# The estimation of metaloproteinases and their inhibitors blood levels in patients with pancreatic tumors

**DOI:** 10.1186/1477-7819-11-137

**Published:** 2013-06-14

**Authors:** Jacek Śmigielski, Łukasz Piskorz, Renata Talar -Wojnarowska, Ewa Malecka-Panas, Sławomir Jabłoński, Marian Brocki

**Affiliations:** 1 Department of Thoracic, General and Oncological Surgery, Medical University, 113 Zeromskiego Street, 90-549 Lodz, Poland; 2 Department of Digestive Tract Diseases, Medical University of Lodz, Lodz, Poland

**Keywords:** Pancreatic cancer, Pancreatic tumor, MMP-2, MMP-9, TIMP-1, TIMP-2

## Abstract

**Background:**

The aim of the study was to evaluate the concentration of proteolytic enzymes, MMP-2 and MMP-9, and their tissue inhibitors, TIMP-1 and TIMP-2, in the blood of patients with benign and malignant pancreatic tumors.

**Methods:**

MMP-2, MMP-9, TIMP-1, and TIMP-2 were evaluated in the patients with benign and malignant pancreatic tumors before surgery and in the 30-day follow-up. The study covered 134 patients aged 54 to 76 years, who were divided into groups by TNM staging.

**Results:**

Before the operation, the highest mean concentration of MMP-2 was found in patients with unresectable cancer, whereas the highest level of MMP-9 was in patients with resectable cancer. The highest level of TIMP-1 was noted in patients with inflammatory tumors. In 1 month following the operation, the highest level of MMP-2 was also in patients with unresectable cancer and the highest level of TIMP-2 in patients with inflammatory tumors.

**Conclusions:**

The evaluation of the level of the studied cytokines in the pancreatic tumor patients can be diagnostically significant in the differentiation of benign and malignant changes. The changes in the levels of the studied enzymes and their inhibitors can have a prognostic value in the clinical severity of pancreatic cancer.

## Background

Is solid tumor of the pancreas a malignant or benign lesion? This simple question is not easy to answer.

Up to now, the most effective method of treating a pancreatic tumor is its early radical surgical removal. However, the early diagnosis of malignant tumors still remains an unsolved problem, which hampers a successful recovery. Despite perfect diagnostic methods for detecting the lesion as malignant or benign, the 100% certain method which could confirm the malignancy of a tumor before surgery does not exist. If a tumor is diagnosed as not malignant prior to surgery, aggressive surgical treatment, which is usually accompanied with a number of complications, including death, can be avoided [1].

The popularization of fine needle biopsy as a diagnostic tool to differentiate benign and malignant changes was highly emphasized many years ago [2]. However, this method can often provide misleading negative results.

Although X-ray imaging can quite precisely denote the position of a tumor, the question posed in the introduction still remains unanswered. In future, the early differential diagnosis of tumors should be rather connected with specific biochemical and molecular markers.

The pancreas is an endocrine gland, well supplied with blood, which facilitates easy transfusion through the permeable walls of the blood vessels [3]. This blood perfusion is important for proper metabolism and endocrine functions. The process of forming new blood vessels from the already existing vessels is called angiogenesis. This process is often present in physiology as part of tissue healing, development of the placenta and endometrium [4]. Furthermore, it is crucial in the pathophysiology of such diseases as sclerosis [5], arthritis [6], diabetic retinopathy [7], or psoriasis [8]. The role of angiogenesis in the biology of neoplasm is extremely interesting [9]. A search is underway for a substance that can stimulate angiogenesis either directly for example by angiogenic cytokines, or indirectly through such agents as extracellular matrix (ECM) degrading enzymes [10].

Metalloproteinases are proteolytic enzymes responsible for the degradation of ECM proteins and the basal membrane of the vessels. They play a significant part in the development of many neoplastic diseases and diseases of the connective tissues [11]. Two of the most important enzymes from this group are matrix metalloproteinase-2 (gelatinase A) MMP-2 and matrix metalloproteinase-9 (gelatinase-B), whose substrates are collagens type I, IV, V, VII, X, XI, XIV, gelatine, fibronectin, laminin, agrecan, and casein. Due to the diversity of the substances affected by this enzyme, its activity level is responsible for the course of many pathological processes in humans [12]. The substances that hamper angiogenesis are tissue inhibitors of metalloproteinases (TIMPs). TIMPs comprise the family of four proteins: multifunctional compounds which, despite their inhibiting impact on MMPs, play a significant role in maintaining a balance between synthesis and ECM proteins in various physiological and pathological processes. TIMP-1 and TIMP-2 are highly specific to MMP-2 and MMP-9 [13].

In the literature there are few reports on the concentration values of metalloproteinases and their tissue inhibitors in serum of the pancreatic tumor patients [14,15]. Similarly, not many reports describe the concentration of these enzymes and their inhibitors due to the advanced stage of a pancreatic tumor.

The aim of the study was to evaluate the concentration of proteolytic enzymes MMP-2 and MMP-9, and their tissue inhibitors TIMP-1 and TIMP-2 in the blood of patients with benign and malignant pancreatic tumors, as well as providing answers to the following questions:

a. Can any changes in concentration of the studied factors in blood be observed?

b. Can the concentration of these factors in blood be treated as a preoperative differential parameter for benign and malignant pancreatic tumors?

c. Does the concentration of MMPs and TIMPs in blood correlate with one another as well as with the clinical severity of a pancreatic tumor?

d. Does the removal of a pancreatic tumor affect the concentration change of these factors? Can a possible difference in concentrations have an influence on the prognosis of a pancreatic tumor?

## Methods

The study was conducted on 134 Caucasian patients aged 54 to 76 years (mean age, 59 years) with pancreatic tumors (118 men, 16 women), who underwent surgical treatment in the Department of Thoracic Surgery, General and Oncological Surgery, Medical University of Lodz, between the years 20072011. Each studied patient was subject to pancreatic and biliary surgery.

The studied patients were divided into four groups depending on the type of tumor, which was classified through the TNM system (under the 7th edition of the American Joint Committee of Cancer – cancers in groups I-III) before and after surgery on the basis of the perioperative picture, the range of surgery, and follow-up within 1 to 2 years after the procedure:

1. Group I (*n*=29): patients with pancreatic cancer at advanced stages I and II of severity on the day of the operation (surgical resection of the pancreas), no recurrence or metastases to distant organs within 2 years following surgery;

2. Group II (*n*=44): patients with pancreatic cancer at advanced stages III and IV of severity (unresectable tumors - bypass anastomoses and/or laparotomy performed);

3. Group III (*n*=37): patients with pancreatic cancer at advanced stages I and II of severity on the day of the operation, recurrence or metastases to distant organs within 2 years after surgery observed;

4. Group IV (*n*=24): patients with benign inflammatory tumors, not malignant tumors often due to chronic pancreatic inflammation (resections of the pancreas).

On histopathological investigation, pancreatic adenocarcinoma was diagnosed in the patients from groups I to III. The study excluded the patients with other histological forms of the tumor, such as neuroendocrine tumors (GEP-NET) and intraductal papillary mucinous neoplasms (IPMN).

The control group consisted of 30 healthy volunteers, men aged 45 to 68 years (mean age, 56 years) who were randomized from the patients with excluded neoplastic changes and inflammatory processes, and who underwent planned surgery due to non-inflammatory cholecystolithiasis. All of these patients underwent routine bedside examinations (physical, labs) and went through a series of abdominal cavity examinations as well as USG, ERCP, MRCP, and/or MRI.

The mean blood concentrations of MMP-2, MMP-9, and their tissue inhibitors TIMP-1 and TIMP-2 were determined prior to surgery, on the 7th and 30th day of follow-up. Blood was obtained by standard blood tests. It was centrifuged and stored at -60°C. The patients were fasting at the time of sampling. The MMP-2 and MMP-9 level in blood was determined using the standard immunoenzymatic ELISA method by means of Amersham Biosciences (sensitivity <0.37 ng/mL; intraserial accuracy (CV) 5.6%; and interserial accuracy (CV) 10%). The TIMP-1 and TIMP-2 level in blood was determined using the standard immunoenzymatic ELISA method by Amersham Biosciences (sensitivity <3.0 ng/mL; intraserial accuracy (CV) 3.9%; and interserial accuracy (CV) 4.8%).

To determine the optical density of each well immediately, a microplate reader set to 450 nm was used. If the wavelength correction was available, it was set to 540 nm or 570 nm. If the wavelength correction was not available, readings were subtracted at 540 nm or 570 nm from the readings at 450 nm. This subtraction was corrected for optical imperfections in the plate by means of Metertech 960 (Bellco Biomedica Company).

All patients signed informed consent forms and ethical approval was obtained from the Bioethics Department of the Medical University of Lodz, Poland (reference RNN/54/10/KB).

The statistical analysis was performed with SPSS (Statistical Package for Social Science, version 16.0) for Windows. Data are shown as mean ± standard deviation and 95% confidence intervals where applicable. The mean differences among the groups were evaluated by one-way ANOVA. *P* values <0.05 were considered statistically significant.

The work was financed under the Medical University own study no. 502-03/5-138-01/502-54-017.

## Results

The study has evaluated the active forms of MMP-2 and MMP-9. The highest mean concentration of MMP-2 before surgery was in group II - 1,347.12 ± 168.6 (95% CI 1,257.3-1,436.9) ng/mL, whereas the lowest mean concentration was noticed in group I - 1,255.26 ± 140.6 (95% CI 1,187.51,323.0) ng/mL. Although these results were higher than in the control group, they were not statistically significant (*P* >0.05).

**Figure 1:**
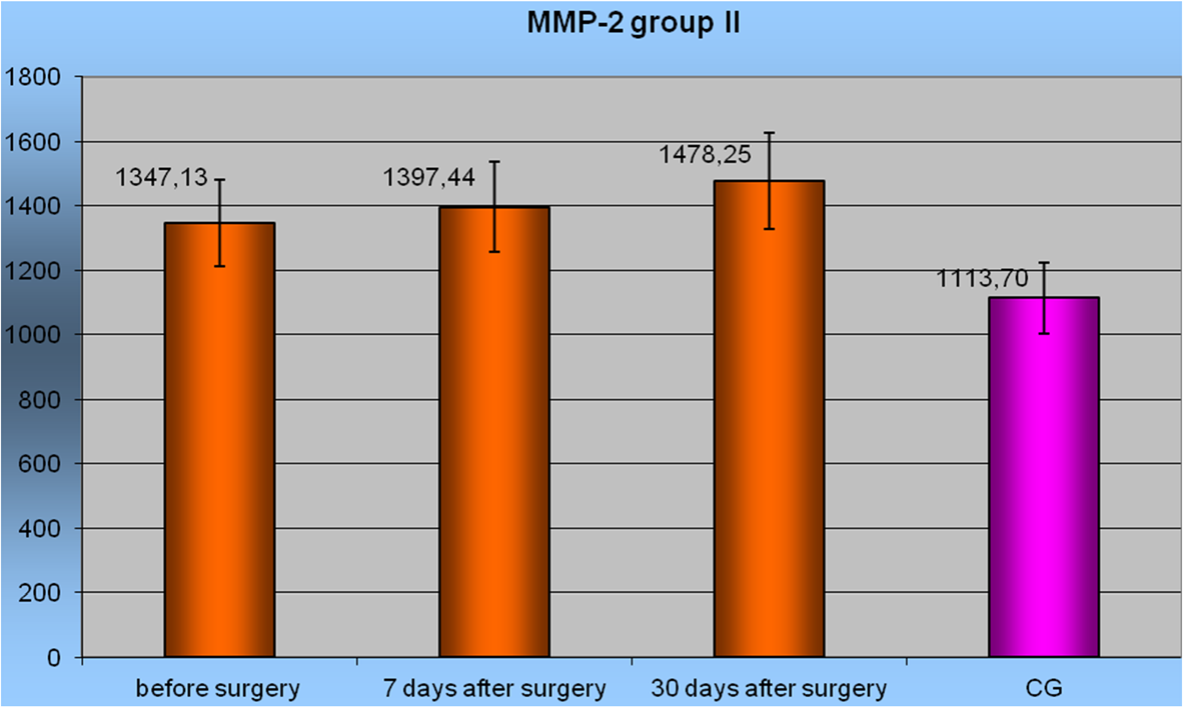
Level MMP-2 in group II before surgery, in 7 and 30 days after surgery to Control Group.

Prior to the operation, as well as in the follow-up 1 week later, no statistically significant differences in the mean concentrations of MMP-2 were found in any of the studied groups when compared to the control group (Figure 1). However, 1 month after surgery, the highest mean concentration of MMP-2 measured in group II was 1,478.25 ± 330.1 (95% CI 1,302.4-1,654.2) ng/mL, which was significantly higher than in the control group and in the other groups (*P* <0.05).

**Figure 2:**
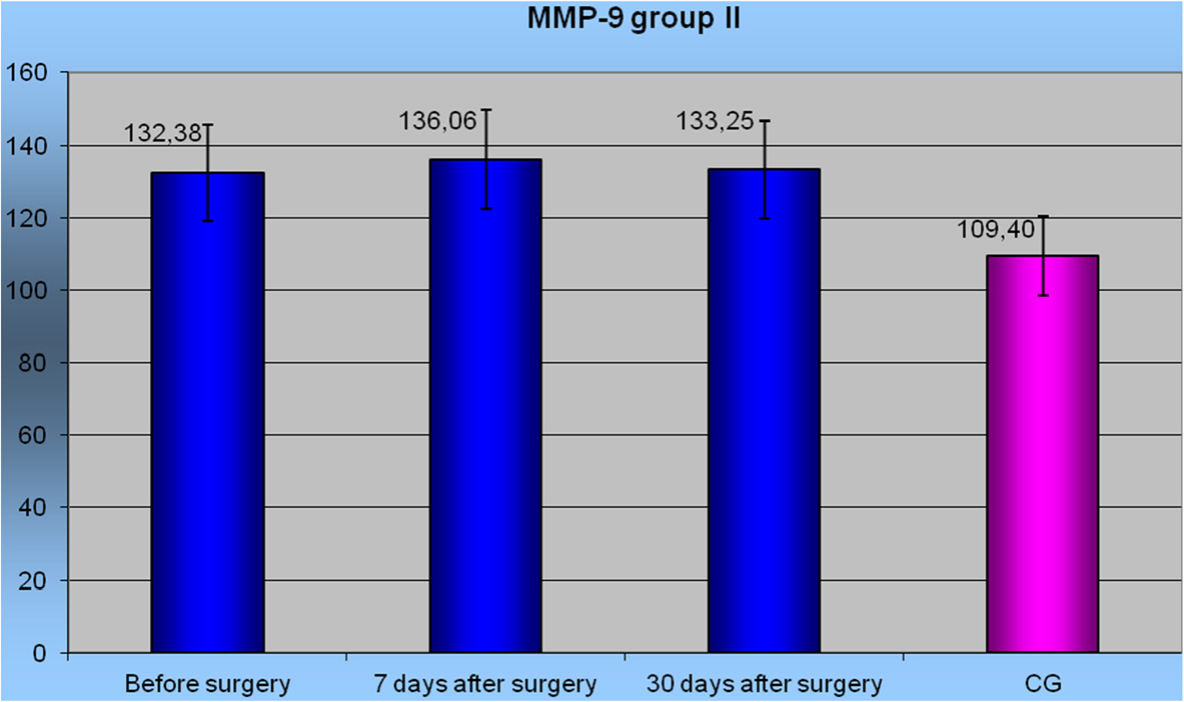
Level MMP-9 in group II before surgery, in 7 and 30 days after surgery to Control Group.

The highest mean concentration of MMP-9 before surgery was in group I: 135.68 ± 43.7 (95% CI 114.6-156.7) ng/mL. The lowest mean concentration was in group IV: 94.07 ± 45.8 (95% CI 67.7-120.5) ng/mL. All the results were significantly higher than in the control group (*P* <0.05). Seven days after surgery the mean concentrations of MMP-9 in groups I, II, and III were significantly higher than in the control group (*P* <0.05). The results between the groups were not significantly different. However, on the 30th day following the operation, the mean concentration of MMP-9 in each group was not significantly different from the control group. No statistically significant differences among the groups were found either. In group II only, the level of MMP-9 was significantly higher than in the control group before and after surgery *P* <0.05 (Figure 2).

**Figure 3:**
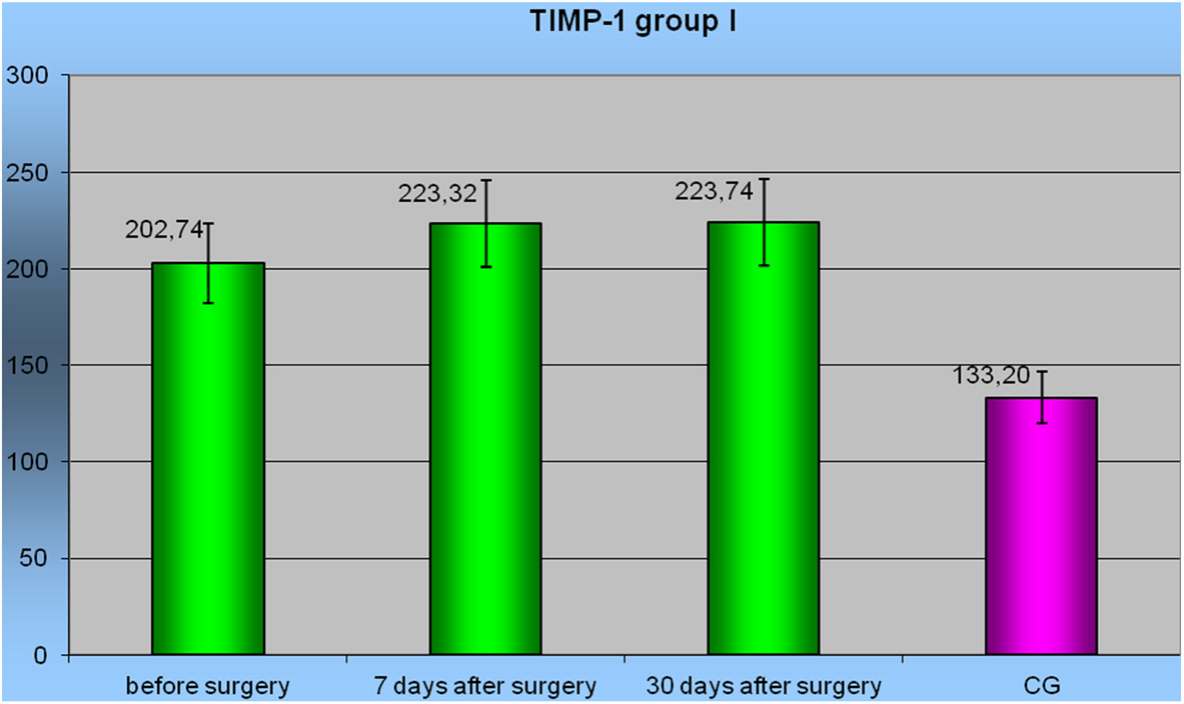
Level TIMP-1 in group I before surgery, in 7 and 30 days after surgery to Control Group.

Before the operation, the mean concentration of TIMP-1 was higher in patients with pancreatic tumors than in the control group (Table 1). The highest mean concentration of TIMP-1 was observed in group IV (207.00 ± 33.4 (95% CI 187.8-226.2) ng/mL) in patients with inflammatory tumors before surgery. The lowest mean concentration was found in group II: 143.31 ± 30.5 (95% CI 127.1-159.6) ng/mL. Only the mean concentrations of TIMP-1 in groups I and IV were significantly higher (*P* <0.05) than in the control group. In a 7-day follow-up, the mean concentrations of TIMP-1 in each group were significantly higher than in the control group (*P* <0.05). Only the mean concentration of TIMP-1 in group II (152.50 ± 34.7 (95% CI 134.0-171.0) ng/mL) was statistically different from the results of the remaining groups (*P* <0.01). Similarly, in the 30-day follow-up (Table 2) the mean concentrations in each group were significantly higher than those of the control group, and the mean concentration of TIMP-1 in group II was significantly different from the mean concentrations in the other groups (*P* <0.05). Before and after surgery, a significantly higher level of TIMP-1 was noticed only in group I *P* <0.05 (Figure 3).

**Table 1:** **Level TIMP-1 and (±) standard deviation by patients with pancreatic tumors in all groups before surgery and statistical significance ( *P *)**

**TIMP-1 (ng/mL)**	**Mean (x)**	**Standard deviation**	***P vs. *CG**
I	202.73	± 74.60	0.015
II	143.31	± 30.52	*P* >0.05
III	189.63	± 45.66	*P* >0.05
IV	207.00	± 33.28	0.008
CG	133.20	± 18.39	X

**Table 2:** **Level TIMP-1 and (±) standard deviation by patients with pancreatic tumors in all groups in 30 days after surgery and statistical significance ( *P *)**

**TIMP-1 (ng/mL)**	**Mean (x)**	**Standard deviation**	***P vs. *GI**	***P vs. *GII**	***P vs. *GIII**	***P vs. *GIV**
I	223.74	± 69.00	X	0.002	*P* >0.05	*P* >0.05
II	141.00	± 32.19	0.002	X	<0.001	<0.001
III	210.68	± 45.18	*P* >0.05	<0.001	X	p>0.05
IV	211.64	± 29.19	*P* >0.05	<0.001	*P*>0.05	X
CG	133.20	± 18.39	<0.001	<0.05	<0.001	<0.001

**Figure 4:**
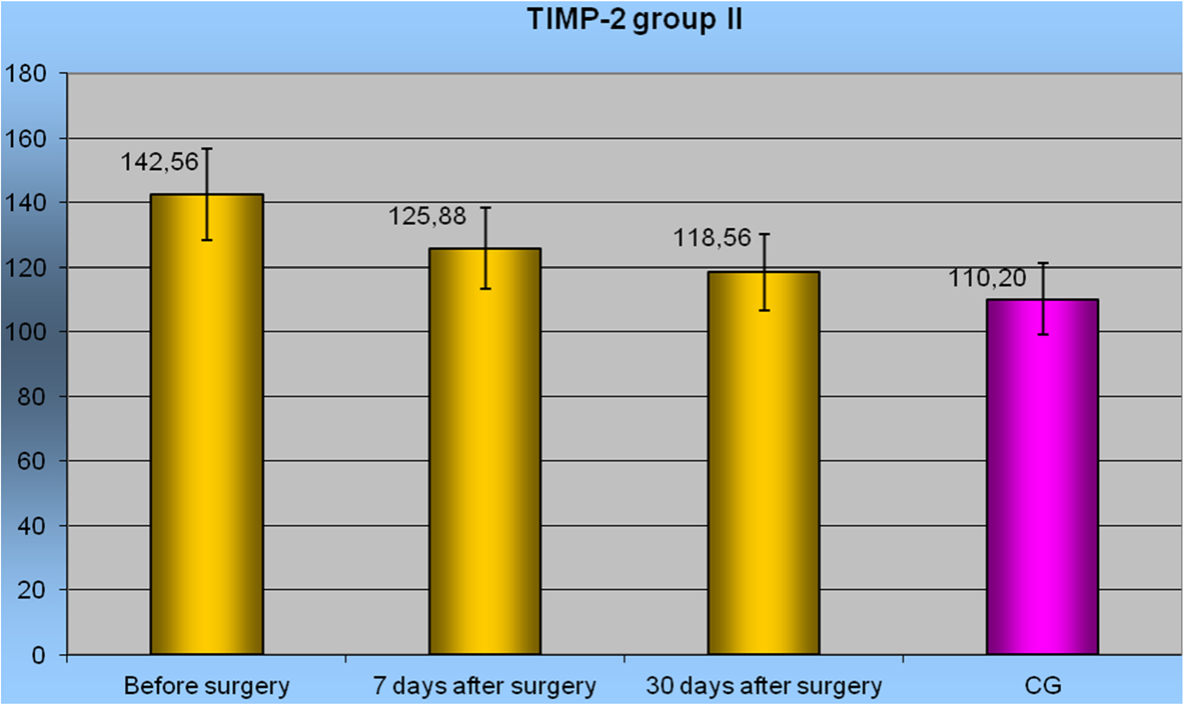
Level TIMP-2 in group II before surgery, in 7 and 30 days after surgery to Control Group.

Prior to surgery, the level of TIMP-2 in the pancreatic tumor patients was higher than in the control group (Table 3). Before the operation, the highest mean concentration of TIMP-2 was observed in group II (142.56 ± 28.8 (95% CI 127.2-157.9) ng/mL) and was statistically significant (*P* <0.05). The mean concentrations of TIMP-2 in the other groups did not differ significantly from those found in the control group. Seven days after the operation, in each group, the mean concentrations of TIMP-2 did not differ significantly from the control group. At the 30-day follow-up, the highest mean concentration of TIMP-2 (158.78 ± 30.3 (95% CI 141.3-176.3) ng/mL) was noticed in group IV. This result was significantly higher than the control group (Table 4) and significantly higher than in the other groups (*P* <0.05). The changes of TIMP-2 level in relation to the control group before and after surgery are depicted in Figure 4.

**Table 3:** **Level TIMP-2 and (±) standard deviation by patients with pancreatic tumors in all groups before surgery and statistical significance ( *P *)**

**TIMP-2 (ng/mL)**	**Mean (x)**	**Standard deviation**	***P vs. *CG**
I	136.53	± 25.85	*P* >0.05
II	142.56	± 28.82	0.03
III	129.21	± 25.70	*P* >0.05
IV	125.71	± 24.27	*P* >0.05
CG	110.20	± 10.15	X

**Table 4:** **Level TIMP-2 and (±) standard deviation by patients with pancreatic tumors in all groups in 30 days after surgery and statistical significance ( *P *)**

**TIMP-2 (ng/mL)**	**Mean (x)**	**Standard deviation**	***P vs. *GI**	***P vs. *GII**	***P vs. *GIII**	***P vs. *GIV**
I	128.95	± 23.82	X	*P* >0.05	*P* >0.05	*P* >0.05
II	118.56	± 19.84	*P* >0.05	X	*P* >0.05	*P* >0.05
III	132.84	± 26.96	*P* >0.05	*P* >0.05	X	*P* >0.05
IV	158.78	± 30.27	0.014	<0.001	0.042	X
CG	110.20	± 10.15	*P* >0.05	*P* >0.05	*P* >0.05	0.005

**Figure 5:**
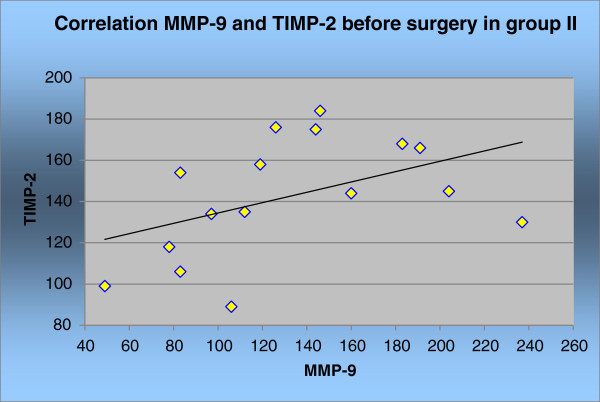
Correlation MMP-9 and TIMP-2 in group II before surgery.

**Figure 6:**
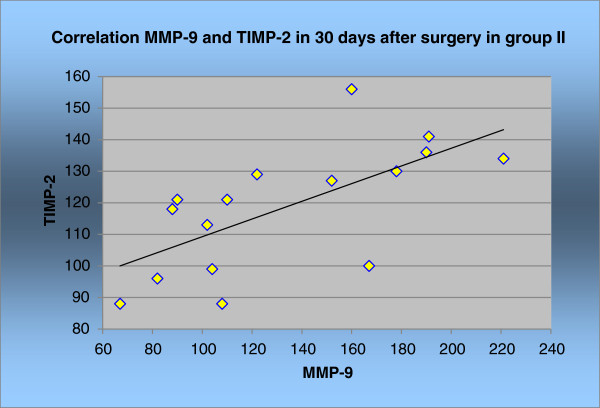
Correlation MMP-9 and TIMP-2 in group II in 30 days after surgery.

The correlation between MMP-9 and TIMP-2 was statistically significant only in group II before, and 30 days after, surgery (*P* <0.05). This is shown in Figures 5 and 6 and Tables 5 and 6.

**Table 5:** Correlation MMP-9 and TIMP-2 in group II and correlation index rang ρ before surgery

**Correlation group II**	**MMP-9 & TIMP-2**
Correlation index rang ρ	0.5
*P*	0.04613
*N*	19

**Table 6:** Correlation MMP-9 and TIMP-2 in group II and correlation index rang ρ in 30 days after surgery

**Correlation group II**	**MMP-9 & TIMP-2**
Correlation index rang ρ	0.76
*P*	0.00059
*n*	19

## Discussion

Pancreatic cancer, its diagnostics, and therapy are the subject of many meta-analyses [16-18]. The dynamics of solid tumor growth often results in a late diagnosis, which, consequently, hampers the radical treatment possibilities. Therefore, the cancer studies largely emphasize the detection of early indicators of oncogenic processes [19-21].

Between 20072011, 181 pancreatic tumor patients were treated in our Department. Surgery was implemented in 153 cases. The rest of the patients were subject to endoscopy, diagnostic laparotomy, or palliative treatment. The work covered 134 patients, out of whom only 29 (21.6%; group I) could undergo therapeutic surgery, whose therapeutic effect was confirmed by a 2-year follow-up without recurrence or metastasis of tumor. The observation of the patients is still ongoing and the complete data can be presented after a 5-year follow-up period.

The role of proteolytic enzymes and their inhibitors has already been affirmed in the growth of many tumors, such as cancer of the esophagus, stomach, large intestine, breast, lung, and thyroid gland [22-26]. However, the role of angiogenesis and the factors participating in the ECM degradation in the pathology of pancreatic tumors is not fully recognized.

The cytokines taking part in ECM degradation, which were observed in the blood of pancreatic tumor patients, are MMP-2 and MMP-9. The study showed that the concentration of MMP-2 was higher in each studied group with reference to the control group, before and after surgery. However, statistically significant growth was found only on the 30th day after surgery in the patients with advanced stages III and IV of pancreatic cancer, who had undergone palliative procedures.

In the evaluation of MMP-9, a significantly higher concentration of this factor was found in the groups with malignant neoplasm, while in the group with benign changes, the concentration of MMP-9 was significantly lower than in the control group. Similar results have been presented by other authors [14,27].

In the evaluation of tissue inhibitors of metalloproteinases, significantly higher mean concentrations of TIMP-1 were observed before the planned surgery in the groups with benign changes or lower advanced stages of pancreatic cancer. Seven and 30 days following the tumor excision, the significantly higher concentrations were still present. In group II of the patients with highly advanced inoperable cancer, no statistically significant differences from the control group were found. However, the statistically significant growth of the mean concentration of TIMP-1 occurred after surgery in the patients with resectable tumors, where recurrence or metastases were noticed.

In the analysis of the next anti-angiogenic factor, TIMP-2, the mean concentration of this cytokine was higher in each group and at each of the three time periods when compared with the control group. A significantly higher level of this inhibitor can be very meaningful in the patients with unresectable cancer of the pancreas before surgery. In the remaining groups, before the operation, no changes in the concentration of TIMP-2 were found.

The TIMP expression is not very well investigated in neoplastic diseases. Nowadays the activity of TIMPs is vital in pathogenesis of the cancer of the esophagus, stomach, intestine, and lungs [28]. Up to now, however, the role of TIMP-1 as a predictive factor has been confirmed only in the case of cancer of the esophagus and the stomach [29].

In lung cancer, a detailed analysis of MMPs and TIMPs was presented by Iniesta *et al*., who demonstrated that MMP-9 can be used as a factor in potentially unfavorable clinical evolution and TIMP-1 bears features of an independent prognostic factor in lung cancer [30]. The evaluation of the studied cytokines may facilitate their future use as neoplastic markers, which, in turn, could allow the advanced stage of pancreatic cancer to be determined before surgery and prognosticate the course of the disease.

The knowledge of whether the concentration and/or activity of these studied factors in blood may be treated as differential parameters in benign and malignant pancreatic tumors before surgery could help surgeons to assess their patients preoperatively. In the future, this might contribute to an early diagnosis and more successful treatment of a pancreatic cancer.

## Conclusions

1. The mean concentration of MMP-2 and MMP-9 in blood of pancreatic cancer patients is significantly higher than in the control group.

2. Before surgery, a significantly higher mean concentration of MMP-9 is seen in the serum of the patients with pancreatic cancer than that of healthy people, and the significantly lower concentration of this enzyme in patients with inflammatory pancreatic tumors, could be diagnostically meaningful in the differentiation of benign and malignant changes.

3. The significantly higher mean concentration of TIMP-1 in blood of the patients with pancreatic tumors at the low advanced stages and significantly higher mean concentration of TIMP-2 in blood of the patients from the other groups and the control group could have a prognostic value in this disease entity.

4. The reciprocal strong positive correlation of MMP-9 and MMP-2 before and after surgery can be significant in the prognosis of the neoplastic advanced stage.

## Competing interest

The authors declare that they have no competing interests.

## Authors’ contributions

ŚJ and T-WR wrote the manuscript, PŁ analyzed the clinical data, EM-P and JS and BM designed the study. All authors read and approved the final manuscript.
